# *Cydonia oblonga* Mill. Whole-Fruit Extract as a Multifunctional Cosmetic Ingredient: Biocompatibility and Concentration-Dependent Effects on the Rheological and Sensory Properties of O/W Emulsions

**DOI:** 10.3390/plants15192958

**Published:** 2026-09-28

**Authors:** Ana Adamovic, Miljan Adamovic, Jovana Dimitrijevic, Marijana Andjic, Marina Kostic, Andjela Gogic, Sanja Matic, Tijana Markovic, Milica Markovic, Dejan Baskic, Jelena Zivkovic, Marina Tomovic

**Affiliations:** 1 Pharmacy Institution “Zdravlje Lek”, Prvomajska 100, 11000 Belgrade, Serbia; 2 Faculty of Medical Sciences, University of Kragujevac, 34000 Kragujevac, Serbia; 3 Department of Pharmacy, Faculty of Medical Sciences, University of Kragujevac, 34000 Kragujevac, Serbia; 4 Center of Excellence for Redox Balance Research in Cardiovascular and Metabolic Disorders,34000 Kragujevac, Serbia; 5 Department of Pharmacology and Toxicology, Faculty of Medical Sciences, University of Kragujevac, 34000 Kragujevac, Serbia; 6 Center for Harm Reduction of Biological and Chemical Hazards, Faculty of Medical Sciences, University of Kragujevac, 34000 Kragujevac, Serbia; 7 Department of Medical Statistics and Informatics, Faculty of Medical Sciences, University of Kragujevac, 34000 Kragujevac, Serbia; 8 Institute for Medicinal Plants Research “Dr. Josif Pancic”, 11000 Belgrade, Serbia

**Keywords:** *Cydonia oblonga*, topical formulations, O/W emulsion, cosmetic emulsions, rheology, sensory evaluation, antioxidant activity, phenolic compounds, biocompatibility

## Abstract

**Background**: *Cydonia oblonga* Mill. is a promising plant-derived ingredient for topical formulations due to its phytochemical composition and potential biological and technological properties. This study aimed to evaluate *C. oblonga* extract as a multifunctional cosmetic ingredient and investigate its concentration-dependent effects on the physicochemical, rheological, and sensory properties of oil-in-water (O/W) emulsions. **Materials and Methods**: Aqueous and 70% ethanolic extracts were prepared by maceration and reflux extraction and evaluated for phytochemical composition and antioxidant activity. The selected extract was assessed for in vitro biocompatibility and antibacterial activity and incorporated at increasing concentrations (1%, 3%, and 5%) into O/W emulsion creams prepared by a conventional hot emulsification method, involving separate heating of the oil and aqueous phases, followed by their combination under mechanical stirring and subsequent addition of the extract during cooling. The formulations were evaluated for pH, steady-shear, oscillatory and thixotropic rheological properties, and sensory characteristics. **Results**: Ethanolic reflux extraction yielded the highest total phenolic and flavonoid contents and the strongest DPPH radical-scavenging activity, with chlorogenic acid identified as the predominant phenolic compound. The extract maintained cell viability above 70% in HaCaT keratinocytes and MRC-5 fibroblasts and showed concentration-dependent antibacterial activity. Increasing extract concentration lowered formulation pH and increased structural strength and resistance to deformation, while all formulations retained shear-thinning behavior. Sensory evaluation revealed significant differences in thickness, stickiness, and spreadability. **Conclusions**: *C. oblonga* extract demonstrated favorable biocompatibility and multifunctional technological potential for the development of topical semisolid formulations.

## 1. Introduction

Topical semisolid formulations represent one of the most frequently used dosage forms in dermal and cosmetic applications due to their ability to deliver active compounds directly to the skin surface while providing protective, moisturising and sensory effects [1]. The performance of these formulations is largely determined by their physicochemical and rheological properties, which directly influence product stability, spreadability, skin retention and overall quality [2]. Rheological behaviour is particularly important during manufacturing, storage, dispensing, application and post-application film formation [3]. Therefore, the development of stable semisolid formulations increasingly focuses on the incorporation of multifunctional ingredients capable of simultaneously improving formulation stability, texture and functional performance [4]. Among topical semisolid systems, oil-in-water (O/W) emulsions are particularly relevant for topical applications because they provide a versatile vehicle for incorporating formulation components of different physicochemical properties while allowing the development of rheological and application characteristics suitable for skin use [1]. In the present study, an O/W emulsion system was therefore selected as an appropriate model for evaluating the incorporation of *C. oblonga* whole-fruit extract and its influence on the structural, rheological and sensory properties of a topical cosmetic formulation.

Plant-derived extracts have attracted considerable attention as natural sources of bioactive compounds and as functional excipients capable of modifying the physicochemical and structural properties of topical formulations [5]. In recent years, increasing attention has been directed towards the incorporation of plant-derived materials into topical formulations owing to their multifunctional biological and technological properties. Among these natural materials, *Cydonia oblonga* (*C. oblonga*) Mill. (quince) represents a promising but still insufficiently investigated natural material for dermal formulations. Different parts of the quince fruit contain phenolic compounds, organic acids, polysaccharides and mucilaginous constituents, which may contribute to both its biological activity and its technological functionality [6]. Phenolic compounds are primarily associated with antioxidant activity, whereas organic acids may influence formulation pH. In addition to their biological activity, phenolic constituents may participate in hydrogen bonding and other non-covalent interactions with formulation components. Together with polysaccharide and mucilaginous constituents, these compounds may influence the continuous phase and interfacial organisation of emulsion systems, thereby modifying viscosity, viscoelastic behaviour and structural organisation. Such modifications may also be reflected in textural and sensory properties, which are important determinants of the acceptability of topical products [7].

Beyond their biological activity, plant-derived ingredients may also modify the technological and application properties of topical semisolid formulations, supporting their use as multifunctional formulation components rather than solely as sources of bioactive compounds [8]. Texture and tactile perception are important determinants of consumer acceptance of topical products and may influence product preference independently of the expected biological benefits. Sensory evaluation provides direct information on how a formulation is perceived before, during and after application; however, the obtained responses may be affected by interindividual variability, skin condition, assessor experience and environmental factors [7,9]. Instrumental rheological measurements provide a complementary and more objective approach for characterising formulation behaviour under conditions relevant to handling and application [10,11]. Steady-shear, oscillatory and thixotropic measurements provide information on resistance to flow, viscoelastic balance and structural breakdown under applied stress [12]. These parameters may show qualitative agreement with sensory attributes such as thickness, spreadability and stickiness, although instrumental measurements cannot fully reproduce the complex perception of a formulation on the skin [13].

Previous original studies have investigated selected applications of *C. oblonga*-derived materials in emulsion-based systems, but with substantially different objectives from those of the present study. A topical emulgel containing *C. oblonga* fruit extract has previously been evaluated primarily with respect to its in vivo effects on skin erythema, sebum, moisture and elasticity, using a single extract-loaded formulation compared with a control [14]. In a different context, seed gum was investigated as a hydrocolloid for stabilizing model O/W emulsions, focusing on emulsion stability, rheology and microstructure [15]. Thus, previous original studies have demonstrated either the biological/cosmetic effects of a quince-containing topical formulation or the technological functionality of isolated quince seed-derived hydrocolloids. However, they did not systematically investigate how increasing concentrations of a whole-fruit extract influence the physicochemical, steady-shear, oscillatory, thixotropic and sensory properties of a topical O/W emulsion system.

The present study addresses this specific gap by incorporating *C. oblonga* whole-fruit extract, including peel, pulp and seeds, at increasing concentrations (1%, 3% and 5%) into O/W emulsion creams and comparing them with an extract-free formulation. In addition to phytochemical characterization, antioxidant activity, cytocompatibility and antibacterial assessment, the study systematically evaluates concentration-dependent changes in formulation pH, steady-shear behavior, oscillatory viscoelasticity, thixotropy and sensory characteristics. This approach therefore extends previous original research by examining the concentration-dependent technological role of *C. oblonga* whole-fruit extract within a topical emulsion system.

## 2. Results

### 2.1. Phytochemical Characterisation of Quince Extracts

The phytochemical composition of quince extracts obtained using different extraction methods is presented in Table 1.

Chlorogenic acid was identified as the predominant phenolic compound in all investigated extracts, followed by rutin and caffeic acid. Hyperoside and nicotiflorin were detected at considerably lower concentrations, whereas isochlorogenic acid A was present in most extracts and detected only in trace amounts in the aqueous macerate. In general, ethanolic extracts contained higher concentrations of the identified phenolic compounds than aqueous extracts. The highest chlorogenic acid concentration was determined in the ethanolic macerate, whereas the highest caffeic acid concentration was observed in the ethanolic reflux extract.

The total phenolic content (TPC), total flavonoid content (TFC), and antioxidant activity of the investigated extracts are presented in Table 2. Ethanolic extracts exhibited higher TPC and TFC values than aqueous extracts, irrespective of the extraction method. The ethanolic reflux extract showed the highest TPC (54.77 ± 2.87 mg GAE/g), the highest TFC (21.24 ± 1.12 mg QE/g), and the lowest IC_50_ value (68.43 ± 3.98 μg/mL), indicating the strongest DPPH radical-scavenging activity among the investigated extracts. Based on these findings, the ethanolic reflux extract was selected for the preparation of the O/W emulsion formulations.

### 2.2. pH of the Investigated Formulations

The pH values of the investigated formulations after 7 and 30 days of storage are presented in Table 3. All formulations exhibited slightly acidic pH values throughout the storage period. Increasing quince extract concentration was associated with progressively lower pH values at both evaluation time points. A slight increase in pH was observed in all formulations after 30 days of storage.

### 2.3. Biocompatibility Assessment

The biocompatibility of the selected *C. oblonga* extract was evaluated in HaCaT keratinocytes and MRC-5 fibroblasts using the MTT assay following 24 and 48 h of exposure (Figure 1). According to ISO 10993-5, cell viability values above 70% indicate that the tested material is non-cytotoxic. Cell viability remained above this threshold at all tested concentrations (1.17–600 μg/mL) in both cell lines after 24 and 48 h of treatment.

In both cell lines, treatment with the extract maintained cell viability above the untreated control throughout the investigated concentration range. HaCaT cells exhibited a more pronounced increase in metabolic activity, particularly after 48 h of exposure, whereas MRC-5 fibroblasts showed a more moderate response. No concentration-dependent reduction in cell viability was observed, even at the highest tested concentration (600 μg/mL), indicating a favorable in vitro safety profile of the extract.

As expected, Tween 20^®^, used as the positive control, produced a concentration-dependent decrease in cell viability in both cell lines, confirming the sensitivity of the experimental model (Figure 1).

### 2.4. Morphological Evaluation

Representative bright-field micrographs obtained after treatment with higher extract concentrations are presented in Figure 2, whereas images corresponding to lower concentrations are shown in Supplementary Figure S1. No treatment-related morphological alterations were observed in either HaCaT or MRC-5 cells following 24 or 48 h of exposure. Throughout the investigated concentration range, both cell lines retained their characteristic morphology, with no evidence of cell rounding, detachment, or accumulation of cellular debris, even at the highest tested concentration.

### 2.5. AO/EB Staining

The effects of the selected *C. oblonga* extract on cell viability and membrane integrity were further evaluated by AO/EB dual staining following 24 and 48 h of treatment. Representative fluorescence micrographs and quantitative analysis of the AO/EB fluorescence ratio corresponding to the higher extract concentrations (75–600 μg/mL) are presented in Figure 3, whereas the results obtained at lower concentrations (1.17–37.5 μg/mL) are shown in Supplementary Figure S2. Quantitative analysis revealed variations in the AO/EB fluorescence ratio among the investigated concentrations in both cell lines, with no consistent concentration- or time-dependent trend. These findings were supported by the representative fluorescence micrographs, which showed predominantly AO-positive cells and only occasional EB-positive cells across all treatment conditions.

### 2.6. Antibacterial Activity

The antibacterial activity of the selected *C. oblonga* extract, expressed as the percentage of growth inhibition, is presented in Figure 4. Growth inhibition generally increased with increasing extract concentration, although the magnitude and concentration–response pattern differed among the investigated bacterial strains. Enterococcus faecalis exhibited the highest susceptibility, whereas Pseudomonas aeruginosa was the least susceptible. Visible bacterial growth was observed at all tested concentrations, including the highest concentration of 16 mg/mL. Accordingly, the MIC was >16 mg/mL for all tested strains. Since none of the tested concentrations produced complete growth inhibition, the MBC could not be determined. Overall, the OD600 measurements demonstrated partial, strain-dependent growth inhibition within the investigated concentration range.

### 2.7. Sensory Evaluation of Tested Formulations

The results of the sensory evaluation performed before, during, and after application of the investigated formulations are presented in Table 4, while the overall sensory profiles are illustrated in Figure 5.

Statistically significant differences between formulations were observed for several sensory attributes (Table 4). Before application, significant differences were detected for gloss level (*p* = 0.048) and elasticity (*p* = 0.008). F4 exhibited a significantly lower gloss level than F1 and F2, while F3 did not differ significantly from the other formulations. F1 received significantly higher elasticity ratings than F2 and F4, whereas F3 did not differ significantly from the other formulations.

During application, statistically significant differences were observed for spreadability (*p* = 0.035), stickiness (*p* = 0.003), thickness (*p* < 0.001), and absorption (*p* = 0.043). Formulation F4 was perceived as the thickest and most adhesive formulation, whereas formulation F3 achieved the highest spreadability scores. Post hoc comparisons indicated significantly higher absorption scores for F2 than for F1, whereas F3 and F4 did not differ significantly from the other formulations.

After application, residual coating was the only sensory attribute that differed significantly between formulations (*p* = 0.009). The placebo formulation (F1) exhibited the highest residual coating scores, whereas formulation F3 showed the lowest values for this parameter.

The radar plot presented in Figure 5 summarizes the overall sensory profiles of the investigated formulations and illustrates the differences observed among the evaluated sensory attributes.

No statistically significant differences between formulations were observed in adhesion, texture, greasiness, shine or post-application stickiness (*p* > 0.05; Supplementary Table S1).

### 2.8. Rheological Characterisation of the Tested Formulations

#### 2.8.1. Thixotropic Behavior (Hysteresis Loop Analysis)

The hysteresis loop areas obtained for formulations F1–F4 are presented in Table 5.

The hysteresis loop area values differed significantly between the investigated formulations (one-way ANOVA, *p* < 0.001). Formulation F4 exhibited the largest hysteresis loop area, followed by F2, while formulations F1 and F3 showed lower values and belonged to the same homogeneous subset according to Tukey’s HSD post hoc analysis. The assumption of homogeneity of variances was confirmed by Levene’s test (*p* = 0.066). The larger hysteresis loop area values indicated greater structural breakdown during shear.

Representative hysteresis loops illustrating the thixotropic behavior and structural recovery of formulations F1–F4 are presented in Figure 6.

#### 2.8.2. Amplitude Sweep Analysis

The parameters obtained from amplitude sweep analysis are presented in Table 6.

##### Linear Viscoelastic Region (LVER)

The LVER values of the investigated formulations are presented as mean ± SD (*n* = 3). No statistically significant differences between formulations were observed (*p* > 0.05). Due to heterogeneity of variances confirmed by the Levene test (*p* < 0.05), the Welch robust ANOVA test was additionally applied, which also indicated no statistically significant differences between formulations (*p* = 0.244).

##### Storage Modulus (G′)

Statistical analysis revealed significant differences in G′ values within the LVER between formulations (Welch test, *p* = 0.028). Formulation F4 exhibited markedly higher G′ values compared to the remaining formulations, indicating greater structural strength and resistance to deformation.

##### Flow Point Stress (τ) and Strain (γ)

Flow-point stress (τ) differed significantly between formulations (Welch’s ANOVA, *p* = 0.013). F4 exhibited the highest flow-point stress and a significantly higher value than F1, whereas F2 and F3 did not differ significantly from the other formulations. Although an overall significant difference in flow-point strain (γ) was detected (*p* = 0.049), post hoc analysis revealed no statistically significant pairwise differences between formulations.

#### 2.8.3. Frequency Sweep Analysis

The viscoelastic parameters obtained from frequency sweep analysis are presented in Table 7.

The frequency dependence of the storage modulus (G′) and loss modulus (G″) for the investigated formulations is shown in Figure 7.

##### Storage Modulus (G′)

Frequency sweep analysis revealed significant differences in storage modulus (G′) values between formulations (one-way ANOVA, *p* < 0.001). Formulation F4 exhibited the highest G′ values, while F2 showed higher values compared to F1 and F3, indicating greater structural rigidity and elastic behavior.

##### Loss Modulus (G″)

Significant differences in loss modulus (G″) values were also observed between formulations (one-way ANOVA, *p* < 0.001), with formulation F4 exhibiting the highest values among the investigated samples, indicating a more pronounced viscous component of the emulsion structure.

##### Loss Factor (tan δ)

Formulation F3 exhibited significantly higher tan δ values compared to the remaining formulations, indicating a shift towards more viscous and less elastic behavior.

#### 2.8.4. Flow Behavior and Rheological Modeling

The flow behaviour of the investigated formulations was evaluated using steady-shear measurements and rheological model fitting. The coefficients of determination (R^2^) obtained for the applied rheological models are presented in Table 8, while the corresponding rheological parameters are summarised in Table 9. For F2, all evaluated models showed excellent fitting performance (R^2^ = 0.99). The Cross, Carreau–Yasuda and Carreau models also provided high R^2^ values for F3 and F4, whereas greater variability in model performance was observed for F1.

Lower correlation coefficients were observed for formulation F1, particularly for the Carreau model, whereas the Cross model demonstrated consistently high fitting accuracy across all investigated formulations.

The rheological parameters obtained from steady shear measurements and model fitting are presented in Table 9.

Although several rheological models were evaluated, only the Cross and Ostwald models were selected for detailed presentation because they provided the most relevant parameters for comparative analysis.

Statistically significant differences between formulations were observed for several rheological parameters (*p* < 0.05). F4 and F2 exhibited significantly higher apparent viscosities at 0.1 and 100 s^−1^ than F3, whereas F1 did not differ significantly from the other formulations. F2 exhibited a significantly higher infinite-shear viscosity (η∞) than F4, while F1 and F3 did not differ significantly from the other formulations. F4 showed the highest consistency index (K), with a significantly higher value than all other formulations, whereas F3 showed the lowest numerical value. The flow behaviour index (n) was significantly higher for F4 than for the other formulations; F2 and F3 also showed significantly higher values than F1.

The viscosity profiles of the investigated formulations as a function of shear rate are presented in Figure 8. All investigated formulations exhibited non-Newtonian shear-thinning behavior, characterized by a decrease in viscosity with increasing shear rate.

### 2.9. Qualitative Comparison of Sensory Attributes and Rheological Parameters

To explore whether the sensory perception of the investigated formulations was consistent with their instrumental characteristics, the sensory findings were qualitatively compared with the corresponding rheological parameters. This exploratory comparison is summarised in Table 10.

The qualitative comparison showed that perceived thickness, stickiness and spreadability followed patterns broadly consistent with selected rheological characteristics, whereas no clear correspondence was observed for elasticity, residual coating or absorption.

## 3. Discussion

### 3.1. Biological Properties of C. oblonga Extract

The incorporation of plant-derived extracts into topical formulations requires not only the demonstration of technological functionality but also confirmation of their biological safety. Therefore, the selected *C. oblonga* extract was additionally evaluated with respect to its cytocompatibility using HaCaT keratinocytes and MRC-5 fibroblasts. According to ISO 10993-5, cell viability values exceeding 70% indicate the absence of cytotoxic effects. In the present study, cell viability remained above this threshold across the entire investigated concentration range after both 24 and 48 h of exposure, demonstrating a favorable in vitro biocompatibility profile [16]. The MTT findings were further supported by morphological evaluation, which demonstrated preservation of the characteristic morphology of both cell lines throughout the investigated concentration range. No evidence of cell rounding, membrane disruption or increased cellular debris was observed, indicating that exposure to the extract did not induce detectable structural alterations [17]. Similar observations were obtained by AO/EB staining, where predominantly viable AO-positive cells were detected regardless of extract concentration, confirming preservation of membrane integrity [18].

These findings are consistent with previous reports describing the favorable biological profile of *C. oblonga*, which has been attributed to its high content of polyphenols, flavonoids and other naturally occurring antioxidants [19]. Besides providing antioxidant activity, these compounds have been shown to exert low cytotoxicity towards normal mammalian cells [17,20], supporting the suitability of quince-derived ingredients for dermal applications [14].

The extract also exhibited concentration-dependent antibacterial activity against all investigated bacterial strains, although complete inhibition of bacterial growth was not achieved within the tested concentration range. *E. faecalis* showed the highest susceptibility, whereas *P. aeruginosa* proved to be the most resistant species [21]. Such differences are consistent with the distinct structural characteristics of Gram-positive and Gram-negative bacteria, particularly the protective outer membrane present in Gram-negative microorganisms [22], which may reduce the penetration of phenolic constituents [23,24].

Collectively, the demonstrated biocompatibility and moderate antibacterial activity [25] complement the favorable physicochemical and rheological characteristics observed for the developed emulsion systems. These biological properties further support the potential application of *C. oblonga* extract as a multifunctional ingredient in topical semisolid formulations [14,16] providing not only technological benefits but also additional functional value.

### 3.2. Influence of Quince Extract on Emulsion Structural Organisation

The present findings demonstrate that quince extract influenced the structural properties of the investigated O/W emulsions. Although the rheological response was not strictly proportional to extract concentration, the formulation containing the highest extract concentration (F4) exhibited the greatest structural strength and resistance to deformation. These changes may be associated with quince-derived polysaccharides and phenolic compounds, which could reinforce the continuous phase and promote the formation of a more interconnected structural network. Previous studies have shown that polysaccharide-rich quince fractions can increase continuous-phase viscosity, facilitate droplet–droplet interactions, and contribute to emulsion stability [15]. Although these mechanisms were not directly investigated in the present study, they provide a plausible explanation for the pronounced strengthening effect observed in F4.

Phytochemical characterisation confirmed chlorogenic acid as the predominant phenolic compound, together with rutin, caffeic acid and several minor phenolic constituents, which is consistent with previously reported phytochemical profiles of *C. oblonga* fruits [26]. Ethanolic extracts exhibited higher total phenolic and flavonoid contents and stronger antioxidant activity than the corresponding aqueous extracts. Among the investigated extraction procedures, ethanolic reflux produced the highest total phenolic and flavonoid contents and the strongest DPPH radical-scavenging activity, supporting its selection for incorporation into the O/W emulsions. Similar findings regarding the extraction efficiency of hydroalcoholic systems have been reported for quince fruits [26]. In addition to their antioxidant properties, these phenolic constituents may contribute to intermolecular interactions within the continuous phase and thereby influence the structural organisation of the emulsion.

### 3.3. Relationship Between pH and Rheological Behavior

The pH values of the investigated formulations decreased progressively with increasing quince extract concentration. This trend is most likely associated with the presence of organic acids and phenolic constituents identified during phytochemical characterisation, particularly chlorogenic and caffeic acids, both of which contribute to the weakly acidic nature of the extract. Consequently, formulations containing higher concentrations of quince extract exhibited lower pH values.

All investigated formulations remained within a slightly acidic pH range that is generally considered compatible with the physiological pH of healthy skin. Maintenance of this pH range may support skin barrier function and create less favorable conditions for the proliferation of many microorganisms during storage. Although microbiological stability was not investigated in the present study, previous studies have shown that mildly acidic formulations are generally less favorable for the proliferation of many microorganisms [27]. Formulations with lower pH values, particularly F4, also exhibited higher storage modulus (G′), consistency index (K), and hysteresis loop area values. Changes in pH may alter intermolecular interactions within the continuous phase, thereby influencing the organisation of the viscoelastic network and, consequently, the rheological behavior and structural stability of the emulsion [28].

### 3.4. Rheological Behavior of Emulsion Creams

All investigated formulations exhibited pronounced shear-thinning behavior, which is characteristic of well-structured semisolid emulsions intended for topical application. Such rheological behavior is considered advantageous because the formulations retain high viscosity under low-shear conditions while becoming easier to spread under the mechanical stress applied during skin application. The observed decrease in viscosity with increasing shear rate is consistent with the progressive disruption and alignment of the internal emulsion structure. Similar rheological behavior has previously been reported for semisolid systems containing natural hydrocolloids and plant-derived polysaccharides, where shear-induced rearrangement of the structural network facilitates application [23].

The substantially higher hysteresis loop area observed for formulation F4 may be associated with the greater amount of extract-derived compounds incorporated into the formulation, including quince-derived polysaccharides. Natural polysaccharides are known to increase intermolecular interactions and promote formation of a more interconnected three-dimensional network within the continuous phase. As a result, a larger amount of structural energy is required to disrupt the system during shear, leading to higher hysteresis loop values [29]. Similar effects have been reported for emulsion systems containing hydrocolloid-rich plant extracts, where increased polymer content resulted in more pronounced thixotropic behavior and greater structural breakdown during shear [23,30].

Oscillatory rheological measurements additionally confirmed substantial differences in viscoelastic behavior between formulations. The substantially higher G′ values observed for formulation F4 suggest that the highest extract concentration promoted the development of a stronger and more interconnected viscoelastic network. This effect may be associated with the greater amount of extract-derived compounds and phenolic compounds capable of participating in hydrogen-bond formation and intermolecular interactions within the continuous phase. Accordingly, the elastic component of the emulsion became more pronounced, resulting in greater resistance to deformation. Consistent with the higher G′ values, formulation F4 also exhibited the highest flow point stress, indicating that a greater applied stress was required to initiate structural breakdown and further supporting the presence of a more resistant emulsion network. Simultaneously, F4 exhibited the highest G″ values, indicating that its pronounced elastic structure was accompanied by a substantial viscous component.

The concentration-dependent rheological changes observed after incorporation of *C. oblonga* extract may reflect several concurrent effects within the emulsion microstructure. Increasing extract concentration increases the amount of extract-derived constituents, particularly polysaccharide and phenolic components, present in the continuous aqueous phase. Polysaccharide-rich constituents may increase continuous-phase viscosity and promote intermolecular associations, thereby strengthening the three-dimensional structural network and increasing resistance to flow [29,30]. In addition, interactions between extract constituents and the emulsifier system may modify the organization of the interfacial layer surrounding the oil droplets and consequently influence droplet–droplet interactions and the overall viscoelastic response of the emulsion [29,30]. Phenolic constituents may also participate in non-covalent interactions within the formulation and may influence their distribution between the aqueous phase, oil phase, and oil–water interfacial region. Therefore, the higher viscosity, consistency index, storage modulus and thixotropic response observed particularly for F4 may result from the combined effects of continuous-phase thickening, increased intermolecular interactions and changes in interfacial organization. Since the microstructure and interfacial composition were not directly characterized in the present study, these mechanisms should be regarded as plausible interpretations rather than directly demonstrated molecular interactions.

Formulation F3 exhibited markedly higher tan δ values than the remaining formulations, indicating a shift towards more viscous and less predominantly elastic behavior. This finding suggests that, at the intermediate extract concentration, interactions between extract-derived compounds and the existing emulsion structure may have altered the organisation of the viscoelastic network. Polysaccharides and phenolic constituents may interact with components of the continuous phase and the interfacial film surrounding the oil droplets, potentially resulting in partial weakening or reorganisation of the network. At the highest extract concentration (F4), the system exhibited a stronger and predominantly elastic structure, as reflected by its lower tan δ and substantially higher G′ values [30,31].

### 3.5. Relationship Between Rheological Parameters and Sensory Perception

The greater perception of thickness observed for formulation F4 corresponded closely with its higher storage modulus (G′), consistency index (K), and hysteresis loop area values. These rheological parameters indicate the presence of a stronger and more interconnected internal structure, which was reflected in the sensory perception of increased product body and greater resistance during application. The observed qualitative agreement between instrumental and sensory findings supports the usefulness of rheological analysis for interpreting formulation performance [32].

Formulations F2 and F3 were perceived as more spreadable than F4, despite exhibiting lower viscosity under application-relevant shear conditions. This observation is likely related to their lower resistance to flow during application together with their pronounced shear-thinning behavior. As viscosity decreased under shear stress, the formulations became easier to distribute over the skin surface [33]. These findings demonstrate that greater structural strength does not necessarily translate into improved spreading performance during application.

Formulation F4 exhibited the highest perceived stickiness, which may be associated with its higher extract concentration, increased viscosity and more pronounced thixotropic behavior. The greater amount of extract-derived compounds, potentially including quince polysaccharides, may contribute to stronger interactions within the continuous phase and consequently increase adhesive sensations during application [34]. Similar increases in stickiness have been reported for semisolid systems enriched with natural polysaccharides and hydrocolloids. Although increased stickiness may be beneficial for products requiring prolonged skin residence, excessive stickiness may negatively affect consumer acceptance, depending on the intended application [35].

Although formulation F4 exhibited the highest structural strength and viscoelastic parameters, the most pronounced residual film was observed for formulation F1. This finding may be related to differences in thixotropic recovery behavior after application, while also suggesting that post-application sensory perception depends not only on rheological properties but also on the composition of the continuous phase and interactions between formulation components and the skin surface [36]. Therefore, the formation of a residual coating appears to be influenced by multiple formulation-related factors rather than solely by structural strength. Several sensory attributes, including greasiness, shine and texture, did not differ significantly between formulations. This observation suggests that increasing quince extract concentration primarily modified the structural organisation and mechanical properties of the emulsions without substantially affecting these particular sensory characteristics. Therefore, the extract-induced changes were more evident in sensory attributes showing good agreement with instrumental rheological measurements, particularly thickness, spreadability and stickiness.

Overall, the sensory evaluation results showed qualitative agreement with instrumental rheological measurements [31]. Parameters associated with structural strength and viscoelastic behavior were generally consistent with sensory descriptors related to thickness and stickiness, while formulations characterized by lower viscosity during shear were perceived as more spreadable [37]. However, not all sensory attributes could be directly explained by instrumental rheological parameters, indicating that formulation composition and skin–formulation interactions also contribute to sensory perception [38]. These findings support the use of rheological analysis as a valuable tool for interpreting consumer-relevant sensory attributes during the development of topical emulsion formulations [35].

### 3.6. Practical Implications for Cosmetic Formulation Design

The obtained results have important practical implications for the development of topical emulsion systems containing natural plant extracts. Increasing the concentration of quince extract progressively enhanced the structural strength of the investigated formulations, resulting in higher storage modulus (G′), consistency index (K), viscosity at low shear rates and hysteresis loop area values. Consequently, formulation F4 exhibited the highest resistance to deformation and the most pronounced viscoelastic character, indicating greater structural strength and resistance to deformation under the investigated rheological conditions.

However, superior structural strength was not necessarily associated with the most favorable sensory performance. Formulations F2 and F3 demonstrated better spreadability and a more balanced sensory profile, indicating that moderate extract concentrations may provide a more acceptable compromise between structural strength and ease of application. These findings emphasize that optimization of topical formulations should consider both instrumental and sensory characteristics rather than relying exclusively on rheological parameters [39].

From a formulation perspective, higher extract concentrations may be advantageous for products requiring enhanced consistency, prolonged residence time on the skin and greater structural strength, whereas intermediate extract concentrations may be more suitable for products where spreadability and user acceptability are prioritized [40]. Therefore, the selection of quince extract concentration should be guided by the intended application and desired product performance.

Although the *C. oblonga* whole-fruit extract was characterized by total phenolic content (TPC), total flavonoid content (TFC), and HPLC analysis prior to its incorporation into the O/W emulsions, these parameters were not re-evaluated in the final formulations. Therefore, the retention and stability of the identified phytochemical constituents after incorporation into the emulsion matrix cannot be directly confirmed from the present data. Further studies should include quantitative determination of relevant marker compounds in the finished formulations, as well as their monitoring during storage, to assess the chemical stability of the incorporated extract.

In addition, the physical stability of the developed O/W emulsions was not comprehensively evaluated using dedicated stability protocols such as centrifugation, freeze–thaw cycling, accelerated storage conditions, or quantitative assessment of creaming and phase separation. Although formulation pH was monitored after 7 and 30 days of storage and the rheological properties provided information on the structural characteristics of the emulsions, these measurements alone cannot establish long-term physical stability. Future studies should therefore include systematic physical and accelerated stability testing under different storage conditions to assess phase separation, creaming, resistance to centrifugal stress and freeze–thaw cycles, as well as changes in physicochemical and rheological properties over time.

## 4. Materials and Methods

### 4.1. Materials

The components used in the emulsion cream formulations—olive oil, lingonberry seed oil, cetearyl alcohol, cetearyl olivate (and) sorbitan olivate (Olivem 1000), olive oil PEG-7 esters (Olivem 300), aloe vera juice, hydrolyzed wheat protein, glycerol, caprylic/capric triglyceride, vitamin E (tocopherol) and Euxyl^®^ PE 9010 were supplied by Avena Lab–Farmadria (Vršac, Serbia), Unichem Pharm (Čačak, Serbia) and Mireille Lab (Zagreb, Croatia). Distilled water was produced in-house using a laboratory distillation apparatus at the Faculty of Medical Sciences, University of Kragujevac. All chemicals and reagents used for determination of total phenolic content (TPC), total flavonoid content (TFC), DPPH radical scavenging activity and HPLC analysis were purchased from Sigma-Aldrich (St. Louis, MO, USA).

### 4.2. Plant Material

Mature fruits of *C. oblonga* Mill., originating from the village of Oplanić in the Sumadija region, Serbia, were purchased from a local market in Kragujevac, Serbia, in the autumn of 2025. Whole fruits, including the peel, pulp and seeds, were washed with distilled water, cut into thin slices and dried in a laboratory oven at 40 °C until constant mass was achieved. The dried material was ground using a laboratory mill and stored in tightly closed glass containers, protected from light and moisture, until extraction.

#### 4.2.1. Preparation of Plant Extracts

Four extracts were prepared from the dried and coarsely ground whole fruits of *C. oblonga* Mill. using two extraction techniques—maceration and reflux extraction—and two extraction solvents: distilled water and 70% ethanol (*v*/*v*). The plant material-to-solvent ratio was maintained at 1:10 (*w*/*v*) in all extraction procedures.

#### 4.2.2. Maceraton Extraction

Dried and coarsely ground plant material (50 g) was mixed with 500 mL of either distilled water or 70% ethanol (*v*/*v*) and macerated at room temperature for 48 h with occasional stirring. Following extraction, the mixtures were filtered and concentrated under reduced pressure at 40 °C using a rotary evaporator. The concentrated extracts were subsequently evaporated to dryness in a water bath. The resulting dry extracts were transferred into tightly closed containers and stored at 4 °C, protected from light, until further analysis.

#### 4.2.3. Reflux Extraction

Dried and coarsely ground plant material (50 g) was extracted with 500 mL of either distilled water or 70% ethanol (*v*/*v*) under reflux for 4 h at the boiling temperature of the respective solvent. Following extraction, the mixtures were filtered and concentrated under reduced pressure at 40 °C using a rotary evaporator. The concentrated extracts were subsequently evaporated to dryness in a water bath. The resulting dry extracts were transferred into tightly closed containers and stored at 4 °C, protected from light, until further analysis.

### 4.3. Phytochemical Characterisation

#### 4.3.1. HPLC Analysis

The chemical profile of the extract was determined using high-performance liquid chromatography (HPLC) equipped with a diode-array detector according to the previously published procedure [41]. High-performance liquid chromatography (HPLC) analysis was performed using an Agilent 1260 RR system (Agilent, Waldbronn, Germany) equipped with a diode-array detector (DAD), with spectral acquisition in the range of 190–550 nm. Chromatographic separation was achieved on a Zorbax SB-C18 reversed-phase column (150 × 4.6 mm, 5 μm; Agilent) employing gradient elution with acidified aqueous and organic mobile phases. Mobile phase A consisted of 1% (*v*/*v*) orthophosphoric acid in water, while mobile phase B was acetonitrile. The gradient program was as follows: 0–2.6 min, 90–85% A; 2.6–8 min, 85% A; 8–10.8 min, 85–80% A; 10.8–18 min, 80% A; 18–23 min, 80–70% A; 23–25 min, 70–50% A; 25–27 min, 50–30% A; 27–29 min, 30–10% A; 29–31 min, 10–0% A; and 31–34 min, 0% A. The flow rate was maintained at 0.8 mL min^−1^, the column temperature was kept at 40 °C, and an injection volume of 8 μL was used. Chromatograms were monitored at 260, 280, 320, and 360 nm. Compound identification was accomplished by comparing both retention times and UV–Vis spectral characteristics with those of authentic reference standards. Quantification was performed using external standard calibration curves, and the concentrations were reported as micrograms per gram of dry weight (μg/g DW).

#### 4.3.2. Total Phenolic Content (TPC)

The TPC was determined using the Folin–Ciocalteu method [42]. An aliquot of 200 μL of each extract (20–500 μg/mL) was mixed with 1.0 mL of Folin–Ciocalteu reagent previously diluted tenfold with distilled water. After 4 min, 800 μL of sodium carbonate solution (75 g/L) was added, and the reaction mixtures were incubated for 2 h at room temperature in the absence of light. The absorbance was subsequently recorded at 765 nm using an Agilent Cary UV–Vis spectrophotometer (Agilent Technologies, Santa Clara, CA, USA). Quantification was performed using an external calibration curve prepared with gallic acid (0–100 mg/L; A = 0.10281 × C, r > 0.99), and the results were expressed as milligrams of gallic acid equivalents per gram of dry extract (mg GAE/g DW). All analyses were carried out in triplicate, and the values are presented as mean results.

#### 4.3.3. Total Flavonoid Content (TFC)

TFC was assessed using the aluminum chloride colorimetric method [43]. Briefly, 1 mL of the extract or quercetin standard solution (20–500 μg/mL) was mixed with 200 μL of 10% aluminum chloride, 200 μL of 1 M potassium acetate, and 5.6 mL of distilled water. The reaction mixture was allowed to stand for 30 min at room temperature before measuring the absorbance at 415 nm using an Agilent Cary UV–Vis spectrophotometer (Agilent Technologies, Santa Clara, CA, USA). Flavonoid concentration was calculated from a quercetin standard calibration curve prepared in methanol, and the results were expressed as milligrams of quercetin equivalents per gram of dry extract (mg QE/g DW). All measurements were performed in triplicate, and the results are presented as mean values.

#### 4.3.4. Antioxidant Activity (DPPH Assay)

The free radical scavenging activity of the extracts was assessed using the 2,2-diphenyl-1-picrylhydrazyl (DPPH) assay [44]. For the analysis, 200 μL of the extract or standard solution (20–500 μg/mL of corresponding extraction solvent) was added to 2.0 mL of freshly prepared (0.1 mM) methanolic DPPH solution. The reaction mixtures were incubated in the dark at room temperature for 30 min, after which the absorbance was recorded at 517 nm using an Agilent Cary UV–Vis spectrophotometer (Agilent Technologies, Santa Clara, CA, USA). The control consisted of DPPH solution mixed with the extraction solvent without the sample, while the blank contained the extract solution without DPPH to correct for the intrinsic color of the extracts. The percentage of DPPH radical inhibition was calculated according to the following equation:

% Inhibition = 100 × (Ac − As)/Ac

where Ac represents the absorbance of the control and As the absorbance of the sample. The antioxidant activity was expressed as the IC_50_ value, defined as the concentration of extract required to reduce the initial DPPH radical concentration by 50%, which was calculated by nonlinear regression analysis using OriginPro software 2004 (OriginLab Corporation, Northampton, MA, USA). All measurements were performed in triplicate, and the results are presented as mean values. Ascorbic acid was used as positive control.

### 4.4. In Vitro Cytocompatibility Assessment of the Selected C. oblonga Extract

#### 4.4.1. Cell Culture

The biocompatibility of the selected *C. oblonga* extract was evaluated using non-transformed human lung fibroblasts (MRC-5, ATCC CCL-171) and human keratinocytes (HaCaT, CLS 300493). Cells were cultured in Dulbecco’s Modified Eagle Medium (DMEM) supplemented with 10% fetal bovine serum, 1% penicillin–streptomycin, 1% non-essential amino acids and 2 mM L-glutamine under standard culture conditions (37 °C, 5% CO_2_). Upon reaching approximately 70–80% confluence, cells were subcultured using trypsin–EDTA solution.

#### 4.4.2. MTT Assay

Cell viability was assessed using the MTT assay [45]. MRC-5 and HaCaT cells were seeded into 96-well plates (3 × 10^3^ cells/well) and allowed to adhere for 24 h. Cells were then exposed to ten two-fold serial dilutions of the extract (1.17–600 μg/mL) for 24 and 48 h. Tween 20 was used as a positive cytotoxic control because its membrane-disrupting effects at sufficiently high concentrations allow the detection of a cytotoxic response under the assay conditions. Following incubation, MTT solution (0.5 mg/mL) was added to each well and incubated for 3 h. Formazan crystals were dissolved in dimethyl sulfoxide (DMSO) and absorbance was measured at 570 nm using a microplate reader. Cell-free blanks containing the medium, MTT and the corresponding extract concentration were included for each concentration tested. Absorbance values were corrected using the corresponding blanks, and relative cell viability was calculated as follows: Viability (%) = (ODtreated-ODblank/ODcontrol-ODblank) × 100, where ODtreated and ODcontrol represent the mean optical density of treated and untreated wells, respectively, while ODblank corresponds to the absorbance of cell-free blank containing the extract. Each experiment was performed independently three times in triplicate.

#### 4.4.3. Morphological Evaluation

Cell morphology was evaluated following the same treatment protocol as described for the MTT assay. After 24 and 48 h of exposure, representative bright-field images were acquired using an inverted microscope at 200× magnification [46]. Morphological alterations were assessed qualitatively by comparison with untreated control cells.

#### 4.4.4. AO/EB Staining

Cells were seeded and treated under the same experimental conditions as described for the MTT assay. Following 24 and 48 h of treatment, cell viability and membrane integrity were evaluated using acridine orange/ethidium bromide (AO/EB) dual staining. Immediately before use, AO (10 μg/mL, A1301, Invitrogen, Waltham, MA, USA) and EB (10 μg/mL, J67270.14, Thermo Scientific, Waltham, MA, USA) stock solutions were diluted in phosphate-buffered saline (PBS). The staining mixture was applied directly to the cell monolayer to obtain a final concentration of 1 μg/mL of each dye. Following incubation for 20 min, fluorescence images were acquired using a 2020 S-EYE Setup Microscope camera with S-EYE_Setup software (version 1.6.0.11) mounted on a FLUO500T trinocular inverted fluorescence microscope (Gramma Libero, Ljubljana, Slovenia) at 200× magnification. Three images were acquired from different, non-overlapping fields of view for each sample within each experiment. Fluorescence intensity was quantified using Fiji software (version 2.14.0), a distribution of ImageJ [46]. For each image, the green and red fluorescence channels were separated, and mean fluorescence intensity (MFI) was measured within ten regions of interest (ROIs; 180 × 180 pixels) positioned at identical coordinates in both channels. The green-to-red fluorescence ratio following AO/EB staining was calculated for each ROI, and the ten ROI ratios were averaged to obtain one mean ratio per image. The mean ratios from the three images were then averaged to obtain one value for each sample within an experiment. Each experimental condition was evaluated in three independent experiments. The final results were expressed as the mean ± standard deviation (SD) of the three experiment values.

### 4.5. Antibacterial Activity of C. oblonga Extract

#### 4.5.1. Preparation of Bacterial Inocula

The antibacterial activity of the selected *C. oblonga* extract was evaluated against *Staphylococcus aureus* ATCC 43300, *S. aureus* ATCC 6538, *E. faecalis* ATCC 29212 and *P. aeruginosa* ATCC 27853 using the broth microdilution method according to the recommendations of the European Committee on Antimicrobial Susceptibility Testing (EUCAST). Fresh bacterial suspensions were prepared from 24 h cultures and adjusted to the 0.5 McFarland standard before dilution in Mueller–Hinton broth to obtain the required inoculum concentration.

#### 4.5.2. Determination of Minimum Inhibitory and Bactericidal Concentrations

Antibacterial susceptibility testing was performed in sterile 96-well microplates using two-fold serial dilutions of the extract (0.0625–16 mg/mL). Erythromycin, linezolid, and gentamicin served as positive controls for Gram-positive and Gram-negative bacteria, respectively, while untreated bacterial cultures were used as growth controls. Following incubation for 24 h at 37 °C, the minimum inhibitory concentration (MIC) was defined as the lowest concentration preventing visible bacterial growth. To determine the minimum bactericidal concentration (MBC), aliquots from wells without visible growth were subcultured onto Mueller–Hinton agar and incubated for an additional 24 h. Bacterial growth was additionally quantified by measuring optical density at 600 nm and antibacterial activity was expressed as the percentage of growth inhibition relative to the untreated control. Each experiment was performed independently three times in quadruplicate.

### 4.6. Preparation of O/W Emulsion Creams

O/W emulsion creams were prepared using a conventional hot emulsification method. The oil and aqueous phases were heated separately to approximately 75 °C under continuous stirring. The oil phase was gradually added to the aqueous phase under continuous mechanical stirring until homogenization was achieved.

After cooling to approximately 40 °C, dry quince extract, hydrolyzed wheat protein, vitamin E (tocopherol), and Euxyl^®^ PE 9010 were added to the emulsion. Mixing was continued until complete homogenization was achieved Four formulations (F1–F4) containing different concentrations of quince extract were prepared. The selected extract concentrations were chosen to evaluate concentration-dependent changes over a practically relevant concentration range. Formulation F1 represented the placebo formulation (cream base without extract), while formulations F2, F3, and F4 contained 1%, 3%, and 5% of quince extract, respectively. The qualitative composition of all formulations was identical, with the only difference being the concentration of the incorporated extract.

All prepared emulsions were stored in sealed containers at room temperature for 7 days prior to further physicochemical, rheological, and sensory analysis in order to allow equilibration of the emulsion structure. The quantitative composition of investigated formulations is presented in Table 11.

### 4.7. Sensory Evaluation

Sensory evaluation of the emulsion creams was conducted under controlled laboratory conditions, including ambient lighting, a temperature of 21 ± 2 °C, and relative humidity of 45 ± 3%. Sensory attributes were assessed using a questionnaire adapted from previously published studies on topical semisolid formulations [47]. A total of 20 healthy volunteers (16 females and 4 males), aged between 20 and 40 years, participated in the study. The participants were not trained sensory assessors but were familiarized with the study protocol and evaluation procedure before testing.

The required sample size was determined a priori using G*Power software (version 3.1.9.4) based on a statistically significant difference reported for a sensory parameter in a comparable study on topical formulations. Assuming a significance level of α = 0.05 and a statistical power of 80%, the minimum required sample size was estimated to be 18 participants. To account for possible variability in sensory perception and incomplete assessments, 20 volunteers were included in the study [48].

The inclusion criteria were willingness to participate in the study and the ability to verbally express sensory perceptions. The exclusion criteria included the presence of dermatological disorders, skin irritation, wounds, or known hypersensitivity to any formulation component. Skin type was not considered as an inclusion criterion.

Before testing, all participants underwent a short acclimatization period and were instructed not to use topical products on the test area for 12 h prior to evaluation. Approximately equal amounts of each formulation were applied simultaneously to a separate predefined area on the volar forearms, allowing direct comparison of sensory attributes between samples by the same participant. Sensory evaluation was performed immediately after application and during spreading, while post-application attributes were assessed after 10 min.

All formulations were tested by each participant. Samples were presented in identical, randomly coded containers using a randomized single-blind designed protocol to minimize bias. The assessment was performed using a structured questionnaire divided into three stages: before application, during application and after application. Participants were instructed not to discuss their impressions during testing until completion of the evaluation.

Participants rated the formulations using predefined descriptive categories and/or numerical scales from 1 to 10. The questionnaire applied in the study is presented in Table 12. The sensory evaluation protocol was approved by the Ethics Committee of the Faculty of Medical Sciences, University of Kragujevac, Serbia (Approval No. 09-666/4, 13 February 2025). All participants gave written informed consent before participation.

### 4.8. Rheological Measurements

The rheological properties of the investigated emulsion creams were determined with the use of a rotational rheometer (MCR 120, Anton Paar, Graz, Austria). Measurements were performed using a parallel plate geometry (PP25) with a measuring gap of 1 mm. All measurements were conducted at a controlled temperature of 22 ± 1 °C. Before each measurement, the samples were carefully loaded onto the rheometer plate and allowed to equilibrate briefly before the start of the measurement. All measurements were performed in triplicate.

#### 4.8.1. Amplitude Sweep Measurements

Amplitude sweep measurements were performed to determine the linear viscoelastic region (LVER) and the structural strength of the investigated formulations. Measurements were carried out in oscillatory mode at a constant frequency of 1 Hz (ω = 6.28 rad/s) over a strain range of 0.01–100%. The storage modulus (G′), loss modulus (G″), flow point stress (τ), and flow point strain (γ) were recorded.

#### 4.8.2. Frequency Sweep Measurements

Frequency sweep measurements were performed within the previously determined LVER over an angular frequency range from 0.1 to 10 rad/s. Storage modulus (G′), loss modulus (G″) and loss factor (tan δ) were recorded to characterize the viscoelastic behavior and structural stability of the investigated formulations.

#### 4.8.3. Steady Shear Measurements

Steady shear measurements were performed over a shear rate range of 0.1 to 1000 s^−1^ to characterize the flow behavior of the investigated formulations. The obtained flow curves were fitted using the Cross, Carreau–Yasuda, Carreau and Ostwald (Power-law) mathematical models to determine the zero-shear viscosity (η_0_), infinite-shear viscosity (η∞), consistency index (K), and flow behavior index (n).

#### 4.8.4. Thixotropy Measurements

Thixotropic behavior was evaluated using hysteresis loop analysis. Measurements consisted of an ascending shear rate, a constant high-shear interval and a descending shear rate phase. The hysteresis loop area was used as an indicator of structural breakdown and recovery following shear.

### 4.9. pH Measurement

The pH of the investigated formulations was measured after 7 and 30 days of storage to evaluate short-term pH stability during storage. Measurements were performed at room temperature using a calibrated digital pH meter.

### 4.10. Statistical Analysis

Statistical analysis was performed using IBM SPSS Statistics for Windows, version 25.0 (IBM Corp., Armonk, NY, USA). The normality of distribution of rheological parameters was assessed using the Shapiro–Wilk test, while homogeneity of variances was evaluated using Levene’s test. Differences between formulations were analyzed using one-way analysis of variance (ANOVA). For variables with homogeneous variances, Tukey’s HSD post hoc test was applied. When the assumption of homogeneity of variances was not met, Welch and Brown–Forsythe robust tests were used, followed by Games–Howell post hoc comparisons. Results are presented as mean ± standard deviation (SD). Statistical significance was set at *p* < 0.05.

Sensory evaluation data were analyzed as ordinal repeated-measures data, since all participants evaluated all investigated formulations. Differences between formulations were assessed using the Friedman test, followed by pairwise comparisons with adjustment for multiple testing. Statistical significance was set at *p* < 0.05.

## 5. Conclusions

The present study demonstrated that *C. oblonga* whole-fruit extract possesses antioxidant activity, favorable in vitro biocompatibility and moderate antibacterial activity. Its incorporation into O/W emulsions at concentrations of 1%, 3% and 5% resulted in concentration-dependent changes in formulation pH, rheological behavior and sensory characteristics. All formulations exhibited shear-thinning behavior, while the formulation containing 5% extract generally showed the greatest structural strength. Sensory differences were observed particularly in thickness, stickiness and spreadability.

Overall, the results demonstrate that *C. oblonga* whole-fruit extract affects both the biological and technological characteristics investigated in this study and support its further investigation as an ingredient in topical O/W emulsions. Further studies should include comprehensive physical and accelerated stability testing and evaluation of the stability of phytochemical constituents after incorporation into the final formulations.

## Figures and Tables

**Figure 1:**
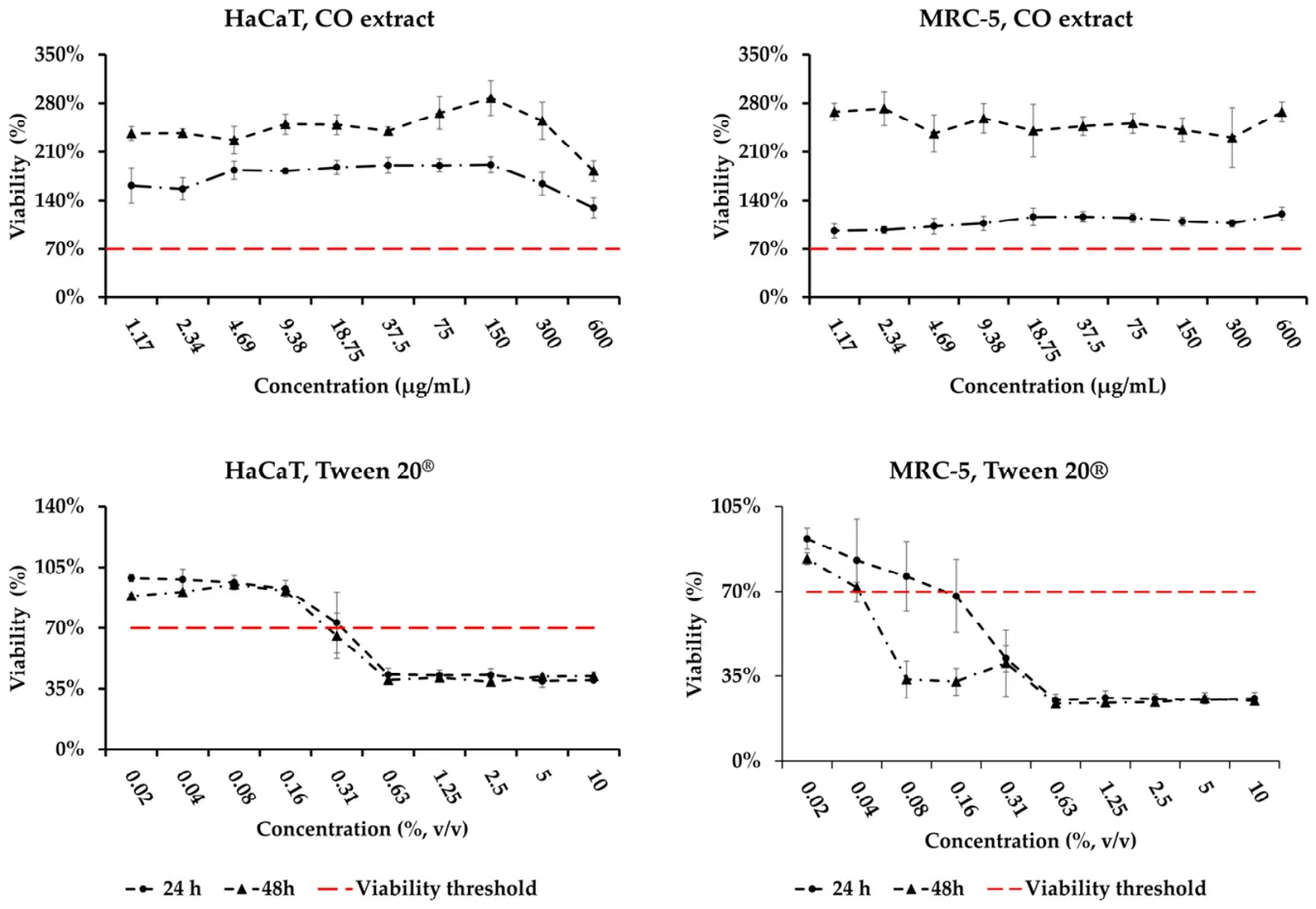
Biocompatibility of *C. oblonga* extract in HaCaT and MRC-5 cells following 24 and 48 h of exposure. Effects of CO extract on the viability of HaCaT keratinocytes and MRC-5 fibroblasts after 24 and 48 h of treatment, determined by the MTT assay. Cell viability is expressed as a percentage relative to the untreated control (100%). The dashed horizontal line indicates the 70% viability threshold defined by ISO 10993-5 for classification of materials as non-cytotoxic. Data are presented as mean ± SD from three independent experiments, each performed in triplicate. CO, *C. oblonga*.

**Figure 2:**
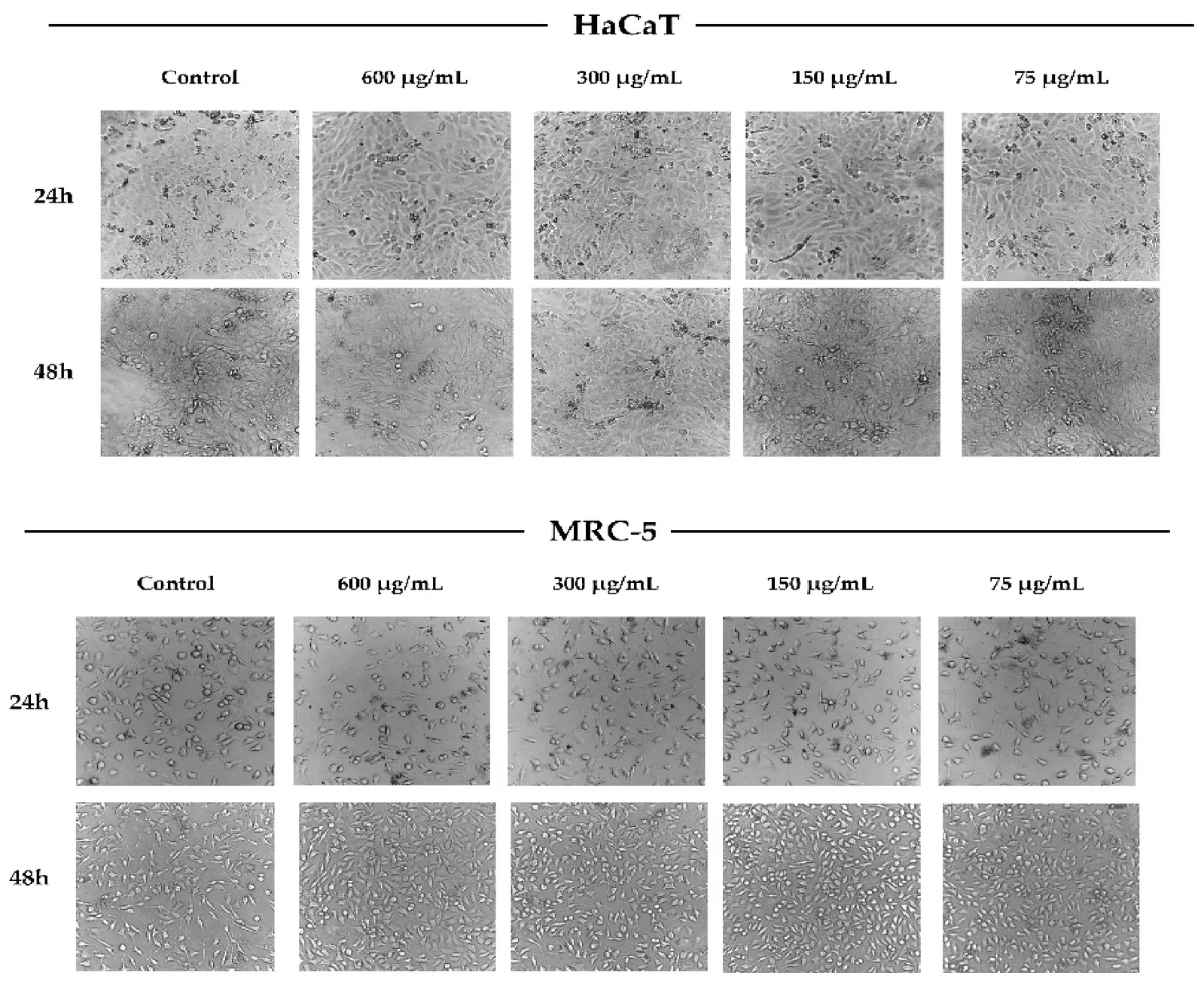
Morphological assessment of HaCaT and MRC-5 cells following treatment with *C. oblonga* extract at higher concentrations. Representative bright-field micrographs of HaCaT keratinocytes and MRC-5 fibroblasts following treatment with CO extract at concentrations of 75–600 μg/mL for 24 and 48 h. Untreated cells served as the negative control. Images were acquired using an inverted bright-field microscope at 200× magnification.

**Figure 3:**
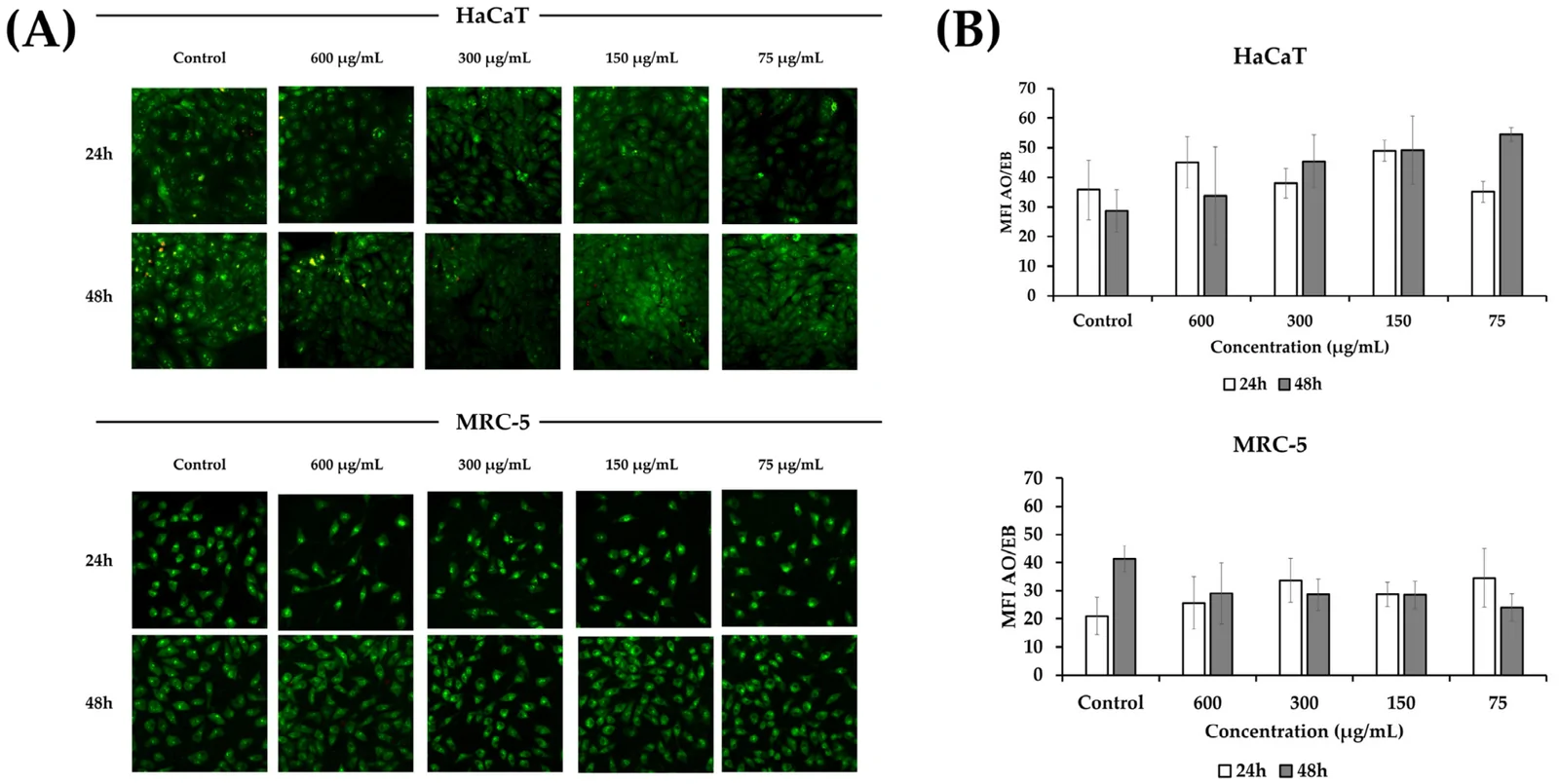
Assessment of cell viability and membrane integrity following treatment with *C. oblonga* extract at higher concentrations. Representative AO/EB fluorescence micrographs (**A**) and quantitative analysis of the AO/EB fluorescence ratio (**B**) in HaCaT and MRC-5 cells following 24 and 48 h of treatment with CO extract at concentrations of 75–600 μg/mL. Images were acquired at 200× magnification. For quantitative analysis, three non-overlapping microscopic fields were analyzed per sample, with ten ROIs evaluated in each field. Fluorescence ratio values are presented as mean ± SD from three independent experiments.

**Figure 4:**
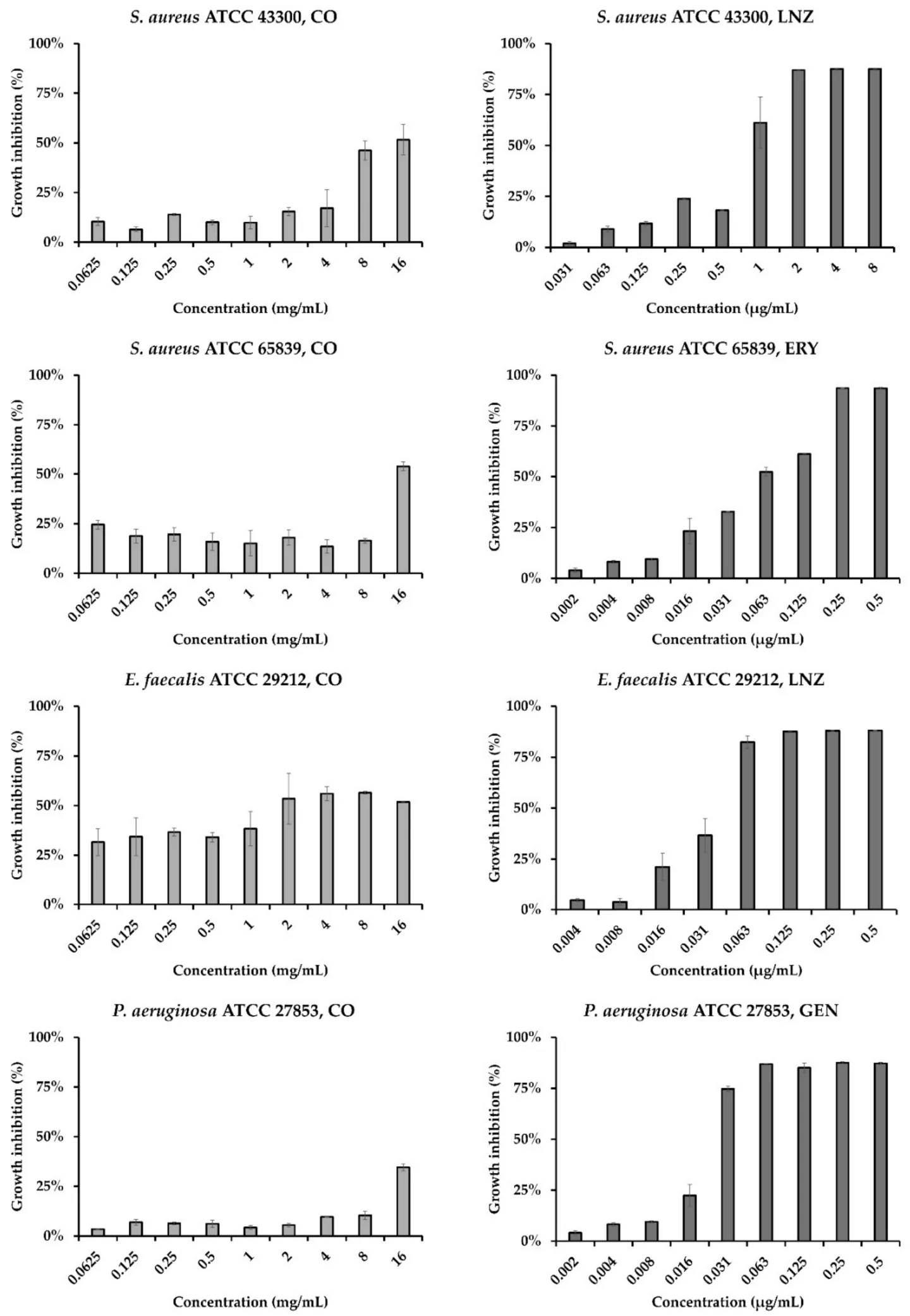
Growth inhibition of reference bacterial strains following treatment with *C. oblonga* extract. Growth inhibition was determined by OD_600_ after 24 h of incubation and expressed as percentage relative to the untreated control. Data are presented as mean ± SD of three independent experiments. CO, *C. oblonga;* ERY, erythromycin; GEN, gentamicin; LNZ, linezolid.

**Figure 5:**
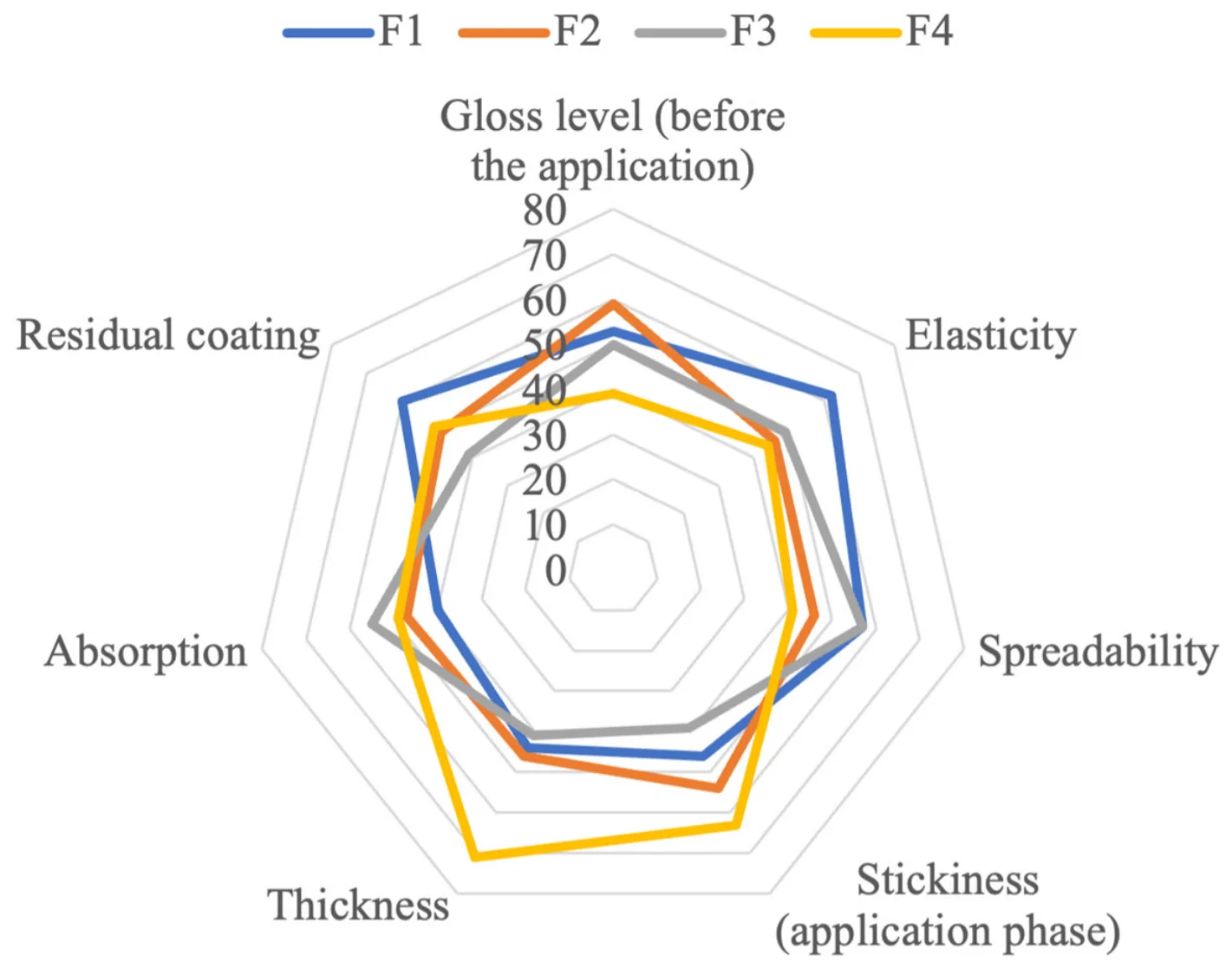
Radar chart illustrating the sensory profiles of formulations F1–F4. Statistical comparisons among formulations and significant pairwise differences for individual sensory attributes are presented in Table 4.

**Figure 6:**
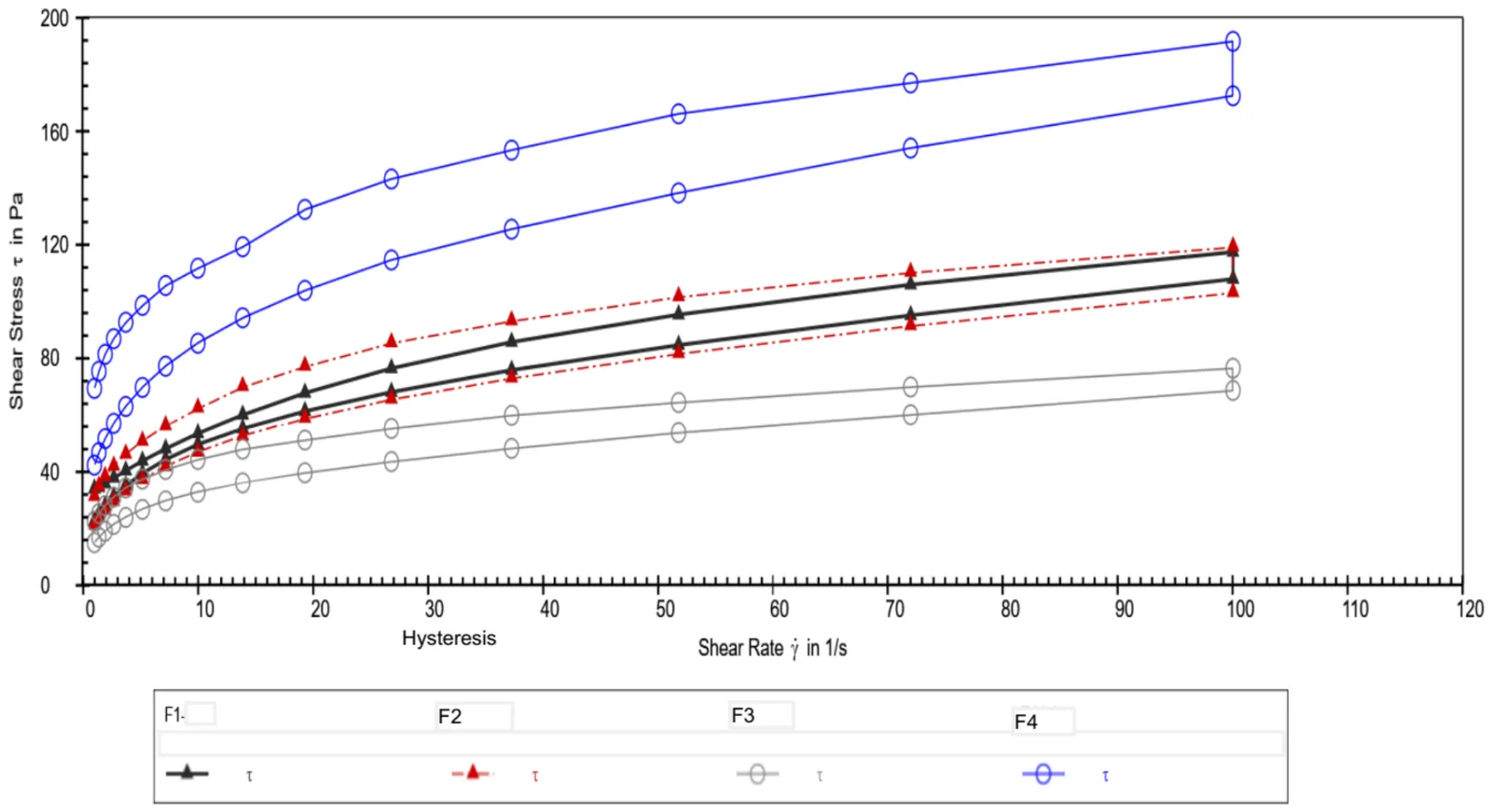
Representative hysteresis loops for emulsion creams F1–F4. Statistical comparisons of the hysteresis loop areas among formulations are presented in Table 5.

**Figure 7:**
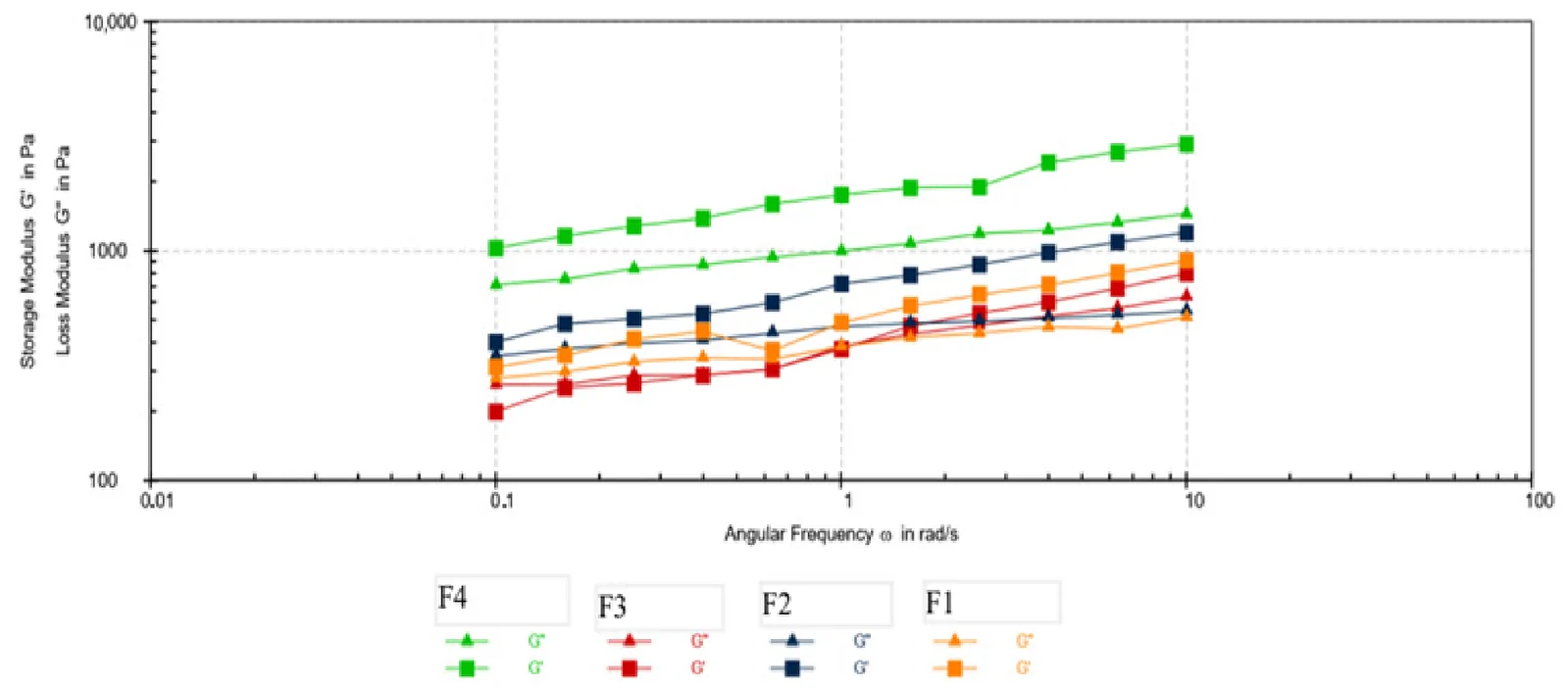
Frequency sweep curves showing storage modulus (G′) and loss modulus (G″) as a function of frequency for formulations F1–F4. Statistical comparisons of the corresponding viscoelastic parameters among formulations are presented in Table 7.

**Figure 8:**
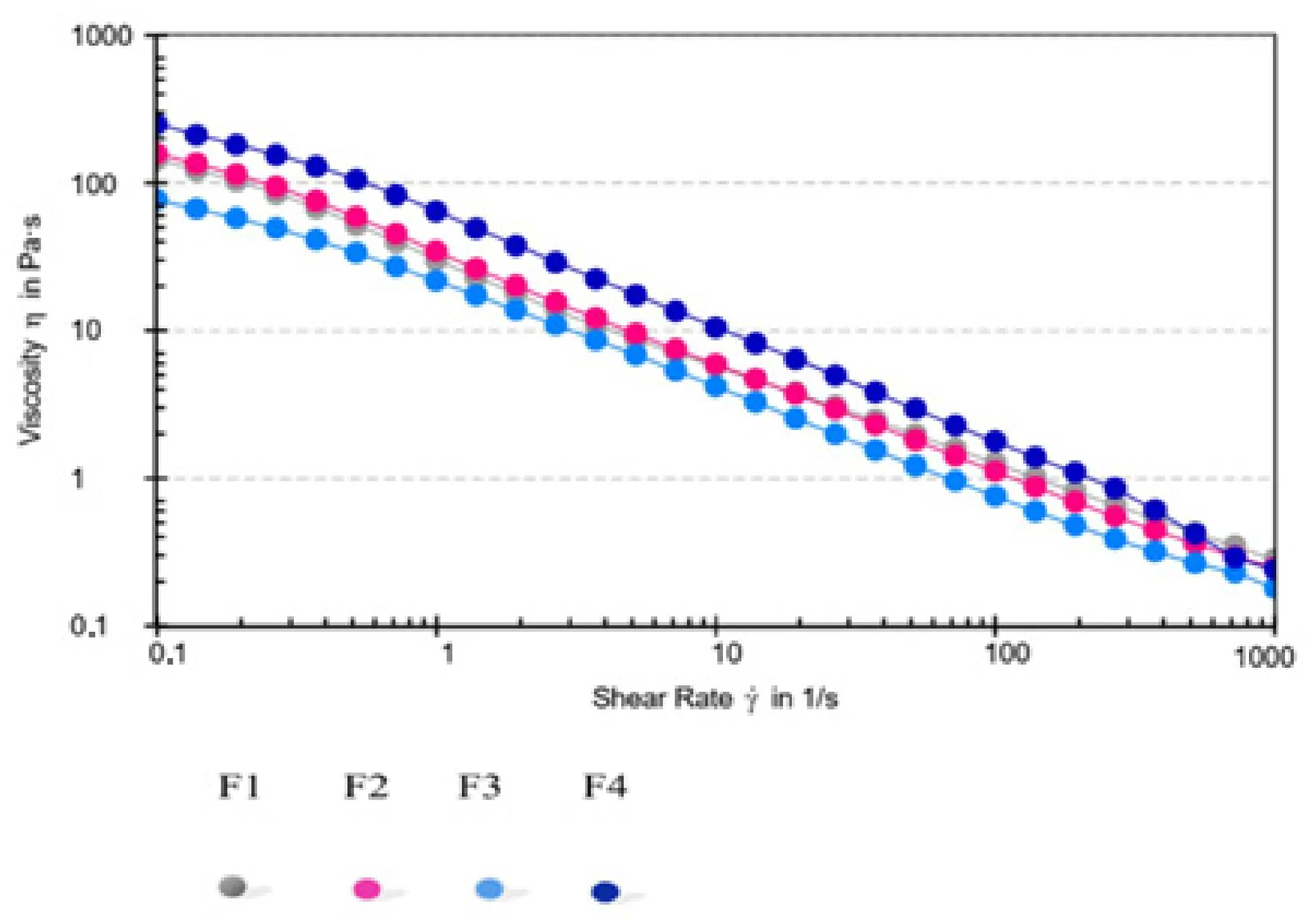
Viscosity profiles of formulations F1–F4 as a function of shear rate. Statistical comparisons of the corresponding rheological parameters among formulations are presented in Table 9.

**Table 1:** Phytochemical composition and antioxidant activity of quince extracts obtained by different extraction methods (mean ± SD).

Compound (µg/g)	Ethanol Reflux	Water Reflux	Ethanol Maceration	Water Maceration
Chlorogenic acid	1356.76 ± 32.15	1196.80 ± 24.22	1395.51 ± 17.89	1171.82 ± 23.98
Caffeic acid	123.85 ± 3.15	89.04 ± 2.18	108.80 ± 2.99	108.78 ± 3.39
Rutin	458.56 ± 11.14	292.72 ± 7.11	503.94 ± 15.93	317.97 ± 10.64
Hyperoside	0.69 ± 0.01	tr	0.78 ± 0.02	0.31 ± 0.00
Isochlorogenic acid A	39.71 ± 1.61	31.96 ± 1.17	37.71 ± 1.82	tr
Nicotiflorin	51.69 ± 1.32	tr	49.64 ± 1.11	17.33 ± 0.48

Values are presented as mean ± SD (*n* = 3). Tr—trace amount.

**Table 2:** TPC, TFC and antioxidant activity (IC_50_) of quince extracts obtained by different extraction methods (mean ± SD).

Parameter	Ethanol Reflux	Water Reflux	Ethanol Maceration	Water Maceration
TPC (mg GAE/g)	54.77 ± 2.87	46.92 ± 3.14	51.72 ± 3.91	43.21 ± 2.63
TFC (mg QE/g)	21.24 ± 1.12	15.67 ± 1.52	19.96 ± 1.71	16.83 ± 1.17
IC_50_ (µg/mL)	68.43 ± 3.98	85.71 ± 4.13	74.38 ± 3.61	93.77 ± 5.18

Values are presented as mean ± SD (*n* = 3). GAE—gallic acid equivalents; QE—quercetin equivalents; IC_50_, concentration required to inhibit 50% of DPPH radicals.

**Table 3:** pH values of the investigated formulations after 7 and 30 days of storage (mean ± SD, *n* = 3).

Time (Days)	F1	F2	F3	F4
7 days	4.39 ± 0.01	4.00 ± 0.01	3.65 ± 0.00	3.47 ± 0.01
30 days	4.57 ± 0.03	4.27 ± 0.02	3.88 ± 0.02	3.7 ± 0.01

Values are presented as mean ± SD (*n* = 3).

**Table 4:** Sensory evaluation results of formulations F1–F4, including rank sums, Friedman test statistics (χ^2^) and corresponding *p*-values.

Sensory Attribute	χ^2^	*p*	F1	F2	F3	F4
Before application						
Gloss level	7.918	0.048 *	53 ^a^	59 ^a^	50 ^ab^	39 ^b^
Elasticity	11.904	0.008 **	62 ^a^	46 ^b^	49 ^ab^	44 ^b^
During application						
Spreadability	8.619	0.035 *	57 ^ab^	46 ^ab^	57 ^a^	41 ^b^
Stickiness	14.058	0.003 **	46 ^ab^	54 ^bc^	39 ^a^	63 ^c^
Thickness	26.651	0.001 **	44 ^a^	46 ^a^	41 ^a^	71 ^b^
Absorption	8.175	0.043 *	40 ^a^	47 ^b^	55 ^ab^	49 ^ab^
After application						
Residual coating	11.469	0.009 **	60 ^a^	49 ^b^	41 ^b^	51 ^ab^

Values reported for F1–F4 represent rank sums. Within each row, formulations sharing at least one superscript letter are not significantly different from each other, whereas formulations with no superscript letters in common differ significantly (*p* < 0.05). For example, a formulation labeled “ab” does not differ significantly from formulations labeled “a”, “b”, or “bc”. * *p* < 0.05; ** *p* < 0.01.

**Table 5:** Hysteresis loop area values (Pa/s) of the investigated formulations after 7 days (mean ± SD, *n* = 3).

Formulation	7-Day Hysteresis Loop
F1	823.46 ± 124.68 ^a^
F2	1888.00 ± 176.00 ^b^
F3	1051.16 ± 60.34 ^a^
F4	2391.40 ± 255.63 ^c^

Values are presented as mean ± SD (*n* = 3). Different superscript letters indicate statistically significant differences between formulations (*p* < 0.05) based on one-way ANOVA followed by Tukey’s HSD post hoc test.

**Table 6:** Viscoelastic parameters obtained from the amplitude sweep analysis of the investigated formulations (mean ± SD, *n* = 3).

Formulation	LVER Limit, γ (%)	G’ Within LVER (Pa)	Flow Point τ (Pa)	Flow Point γ (%)
F1	0.249 ± 0.285 ^a^	880.86 ± 62 ^a^	6.2 ± 10.4 ^a^	1.97 ± 3.22 ^a^
F2	0.357 ± 0.501 ^a^	1178.8 ± 542.9 ^a^	7.49 ± 6.3 ^a,b^	0.835 ± 0.71 ^a^
F3	0.035 ± 0.003 ^a^	990.7 ± 150.8 ^a^	0.6 ± 0.27 ^a,b^	0.054 ± 0.003 ^a^
F4	0.081 ± 0.03 ^a^	3951.66 ± 825.25 ^b^	19 ± 3.6 ^b^	0.941 ± 0.282 ^a^

Values are presented as mean ± SD (*n* = 3). Different superscript letters within a column indicate statistically significant differences between formulations (*p* < 0.05) based on one-way ANOVA with appropriate post hoc tests.

**Table 7:** Parameters obtained from frequency sweep analysis of the investigated formulations.

Formulation	G′	G″	tan *δ*
**F1**	950.17 ± 34.96 ^a^	514.03 ± 19.52 ^a^	0.54 ± 0.03 ^a^
**F2**	1290 ± 98.53 ^b^	689.97 ± 142.87 ^a^	0.53 ± 0.07 ^a^
**F3**	802.07 ± 87.86 ^a^	759.97 ± 138.29 ^a^	0.95 ± 0.14 ^b^
**F4**	2777 ± 132.32 ^c^	1441.67 ± 56.50 ^b^	0.52 ± 0.04 ^a^

Different superscript letters within a column indicate statistically significant differences (*p* < 0.05, Tukey’s HSD test).

**Table 8:** Correlation coefficients (R2) for fitting rheological models to formulations F1–F4.

Sample	Cross Model	Carreau–Yasuda Model	Carreau Model	Ostwald Model
	Correlation Coefficient R^2^			
F1	0.89	0.76	0.66	0.99
F2	0.99	0.99	0.99	0.99
F3	0.99	0.99	0.99	0.98
F4	0.99	0.99	0.99	0.93

**Table 9:** Rheological parameters of the investigated formulations obtained from steady shear measurements and model fitting (mean ± SD, *n* = 3).

Sample			Cross Model Fit	Ostwald Model Fit	
	η (0.1 s^−1^) Pa s	η (100 s^−1^) Pa s	Infinite-Shear Viscosity (η∞) [Pa s]	Consistency Index K (Pa s^n^)	Fluidity Index *n*
F1	135.89 ± 40.34 ^a,b^	1.12 ± 0.14 ^a,b^	0.0402 ± 0.035 ^a,b^	27.66 ± 4.26 ^a,b^	0.35 ± 0.31 ^a^
F2	160.02 ± 7.42 ^b^	1.11 ± 0.02 ^b^	0.0785 ± 0.007 ^b^	33.14 ± 1.12 ^b^	0.73 ± 0.003 ^b^
F3	77.57 ± 8.4 ^a^	0.76 ± 0.06 ^a^	0.0716 ± 0.022 ^a,b^	19.99 ± 1.65 ^a^	0.69 ± 0.001 ^b^
F4	251.13 ± 28.01 ^b^	1.79 ± 0.26 ^b^	0.0011 ± 0.002 ^a^	58.49 ± 7.56 ^c^	0.77 ± 0.003 ^c^

Different superscript letters within a column indicate statistically significant differences between formulations (*p* < 0.05, Games–Howell test).

**Table 10:** Relationship between sensory attributes and corresponding rheological parameters of formulations F1–F4.

Sensory Attribute	Formulation with the Most Pronounced Effect	Related Rheological Parameter(s)	Observed Rheological Characteristic
Thickness	F4	G′, consistency index (K), hysteresis loop area	Higher structural strength and resistance to deformation
Stickiness	F4	Hysteresis loop area, tan δ	Greater structural breakdown during shear and altered viscoelastic behavior
Elasticity	F1	Flow point deformation (γ), LVER	No clear rheological distinction despite differences in sensory perception
Spreadability	F2/F3	Shear-thinning behavior, viscosity at high shear rate	Easier spreading during application
Residual coating	F1	Thixotropic recovery behavior	More pronounced residual film after application
Absorption	F2	Viscosity at high shear rate, tan δ	Modified sensory perception during spreading and absorption. Significantly higher perceived absorption compared with F1

**Table 11:** Composition of investigated emulsion formulations (values expressed as % *w*/*w*).

Phase	Ingredient	F1	F2	F3	F4
Oil Phase	Olive Oil	3	3	3	3
	Lingonberry Seed Oil	5	5	5	5
	Caprylic/Capric Triglyceride	5	5	5	5
	Cetearyl Alcohol	3.5	3.5	3.5	3.5
	Olivem 300	5	5	5	5
	Olivem 1000	8	8	8	8
Aqueous Phase	Aloe Vera Juice	10	10	10	10
	Hydrolyzed Wheat Protein	1	1	1	1
	Glycerol	8	8	8	8
	Vitamin E (Tocopherol)	0.5	0.5	0.5	0.5
	Euxyl PE 9010	1	1	1	1
	Purified Water	ad 100	ad 100	ad 100	ad 100
Active Component	Dry quince extract	0	1	3	5

**Table 12:** Questionnaire applied for sensory attribute evaluation.

Stage	Sensory Attribute	Evaluation Scale
Before application	Consistency	liquid/semisolid
	Gloss level	matte/pearl gloss/slightly glossy/glossy/very glossy
	Adhesion	1–10
	Elasticity	slightly elastic/elastic/very elastic
	Texture	1–10
During application	Spreadability	1–10
	Stickiness	not sticky/slightly sticky/sticky/very sticky
	Thickness	thin/slightly thick/thick/very thick
	Greasiness	not greasy/slightly greasy/greasy/very greasy
	Gloss	not shiny/slightly shiny/shiny/very shiny
	Absorption	slow/moderate/fast
After application	Residual film	no film/moderate/pronounced
	Greasiness	not greasy/slightly greasy/greasy/very greasy
	Gloss	not shiny/slightly shiny/shiny/very shiny
	Stickiness	not sticky/slightly sticky/sticky/very sticky

## Data Availability

The data presented in this study are available in this article.

## Supplementary Materials

The following supporting information can be downloaded at: https://www.mdpi.com/article/10.3390/plants15192958/s1, Figure S1: Morphological assessment of HaCaT and MRC-5 cells following treatment with CO extract at lower concentrations. Figure S2: Assessment of cell viability and membrane integrity following treatment with CO extract at lower concentrations. Table S1: Extended sensory evaluation results for additional attributes of formulations F1–F4 (Friedman test).

## Abbreviations

The following abbreviations are used in this manuscript:

O/W	oil/water
TPC	total phenolic content
TFC	total flavonoid content
GAE	gallic acid equivalents
QE	quercetin equivalents
LVER	Linear viscoelastic region
HPLC	high-performance liquid chromatography
DPPH	2,2-diphenyl-1-picrylhydrazyl
MIC	minimum inhibitory concentration
MBC	minimum bactericidal concentration
