# Supplementary material for: Cydonia oblonga Mill. Whole-Fruit Extract as a Multifunctional Cosmetic Ingredient: Biocompatibility and Concentration-Dependent Effects on the Rheological and Sensory Properties of O/W Emulsions
Source: Plants (Basel). 2026 Sep 28;15(19):2958. doi: 10.3390/plants15192958 (PMC13666955; doi:10.3390/plants15192958)
Supplement: Supplementary file 1 [file plants-15-02958-s001.zip › plants-4520924-supplementary.pdf]

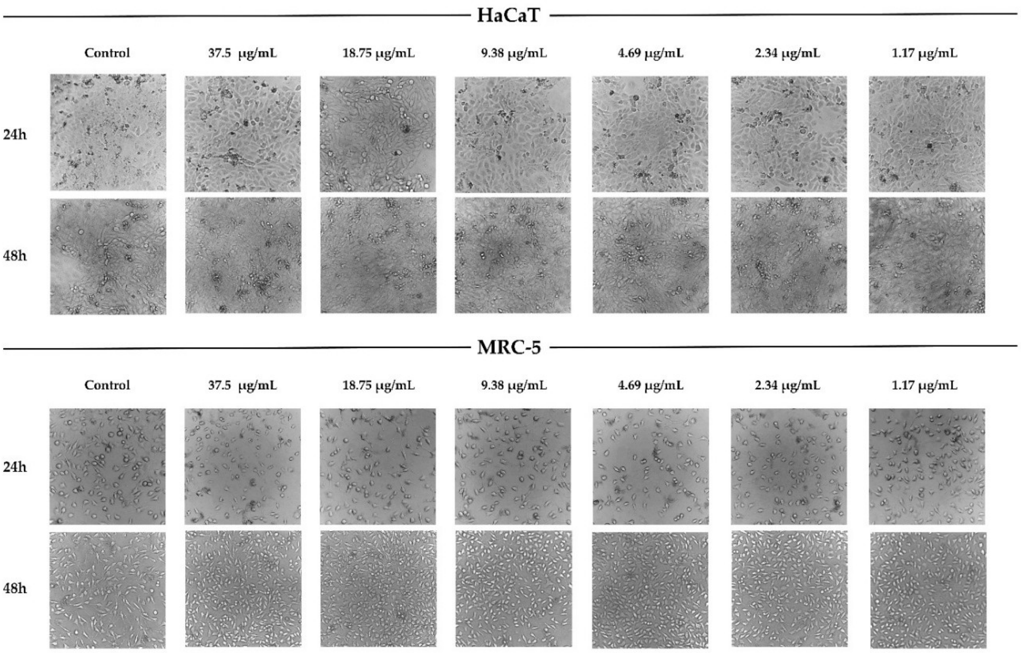

Supplementary Figure S1. Morphological assessment of HaCaT and MRC-5 cells following treatment with CO extract at lower concentrations. The representative micrographs of untreated control cells shown in this figure are intentionally reused, as they represent the same untreated control group presented in the corresponding main figure. The images are repeated for clarity and to facilitate direct comparison with the corresponding treated samples.

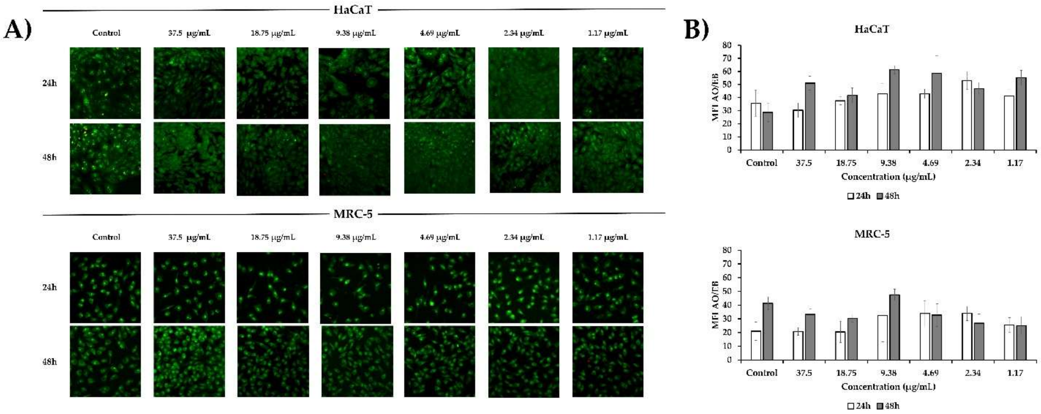

Supplementary Figure S2. Assessment of cell viability and membrane integrity following treatment with CO extract at lower concentrations. The representative micrographs of untreated control cells shown in this figure are intentionally reused, as they represent the same untreated control group presented in the corresponding main figure. The images are repeated for clarity and to facilitate direct comparison with the corresponding treated samples.

Supplementary Table S1. Extended sensory evaluation results for additional attributes of formulations F1–F4 (Friedman test)

Sensory Attribute	χ^2	p	F1	F2	F3	F4
**Adhesion**	3.290	0.349	54	47	45	56
**Texture**	5.337	0.149	45	45	60	52
**Spreadability (secondary)**	4.327	0.228	51	52	56	42

**Greasiness (during application)**	5.731	0.125	50	50	42	58
**Shine (during application)**	7.541	0.057	59	53	44	45
**Greasiness (after application)**	4.230	0.238	53	52	42	53
**Shine (after application)**	7.013	0.071	56	52	49	43
**Stickiness (after application)**	2.551	0.466	47	55	52	47
